# Fit, Precision, and Trueness of 3D-Printed Zirconia Crowns Compared to Milled Counterparts

**DOI:** 10.3390/dj10110215

**Published:** 2022-11-11

**Authors:** Reem Abualsaud, Haidar Alalawi

**Affiliations:** Department of Substitutive Dental Sciences, College of Dentistry, Imam Abdulrahman Bin Faisal University, P.O. Box 1982, Dammam 31441, Saudi Arabia

**Keywords:** 3D printing, yttria-stabilized tetragonal zirconia polycrystals ceramic, dimensional measurement accuracies, dental marginal adaptation, dental internal fit, CAD-CAM, computer-assisted image analyses

## Abstract

Precise fit of a crown and accurate reproduction of the digital design are paramount for successful treatment outcomes and preservation of clinician and technician time. The study aimed to compare the internal fit, marginal adaptation, precision, and trueness of 3D-printed zirconia crowns compared to their milled counterpart. A total of 20 monolithic 3 mol% yttria stabilized-zirconia crowns (*n* = 10) were made using computer-assisted design (CAD) followed by additive (3D-printed) and subtractive (milled) manufacturing. Digital scanning of the master die with and without a fit checker followed by image superimposition, and analysis was performed to evaluate internal and marginal adaptation in four areas (occlusal, axial, marginal, and overall). ISO 12836:2015 standard was followed for precision and trueness evaluation. Statistical analysis was achieved using a *t*-test at α = 0.05. Internal fit and marginal adaptation revealed no significant difference between the two test groups (*p* > 0.05). The significant difference in trueness (*p* < 0.05) was found between the two groups in three areas (occlusal, axial, and internal). The best and worst trueness values were seen with 3D-printed crowns at occlusal (8.77 ± 0.89 µm) and Intaglio (23.90 ± 1.60 µm), respectively. The overall precision was statistically better (*p* < 0.05) in the 3D-printed crowns (9.59 ± 0.75 µm) than the milled (17.31 ± 3.39 µm). 3D-printed and milled zirconia crowns were comparable to each other in terms of internal fit and marginal adaptation. The trueness of the occlusal and axial surfaces of 3D-printed crowns was better, whereas the trueness of fitting surface of milled crowns was better. 3D-printed crowns provided a higher level of precision than milled crowns. Although the internal and marginal fit of both production techniques were comparable, 3D printing of zirconia produced more precise crowns.

## 1. Introduction

Today, monolithic zirconia is a popular material for the fabrication of single crowns, short- and long-span fixed dental prostheses, and full-arch restorations [1,2,3,4,5,6,7,8]. Zirconia has been advocated as a valid treatment option for single-tooth restoration [2,3,4,5,8]. This is owing to its inherent esthetic and mechanical properties [9,10,11].

Zirconia restorations are conventionally produced by a subtractive method (SM), i.e., milling from a blank utilizing computer-aided designing/computer-aided manufacturing (CAD/CAM) [10]. Computerized production from blocks/discs is a reliable process that minimizes human intervention and reduces possible errors seen with the conventional lost-wax technique. The raw material (blocks/discs) can be found in two forms; fully sintered, used for hardmilling, or partially sintered, utilized for green-stage milling. Each of these production paths has its own limitations. Amongst those linked with hard milling are the possible crack formation within the restoration, milling tool wear, lengthy milling time, and surface topographies [12]. At the same time, green-stage milling requires enlarging the design to compensate for sintering shrinkage [13]. In both processing techniques, a large quantity of raw-material waste is anticipated. Additionally, the resulting surface finish and configuration of the restoration are heavily influenced by the geometry and dimensions of the milling tools and the number of milling axes of the machines [10,11,14,15].

A recent advancement in the manufacturing process of dental restorations involves building the object in thin layers of raw material followed by postprocessing curing and finishing steps. This technique is generally called additive manufacturing (AM) or 3D-printing, and it involves different techniques such as thermoplastic extruded material, vat photopolymerization, binder and material jetting, powder bed fusion, and others [15,16]. In dentistry, restorative dentists and lab technicians are using variable AM techniques to produce interim restorations, dentures, casts, and metal prostheses. More recently, there has been an increasing interest in printing ceramic restorations, including zirconia-based prostheses [15,17].

AM has brought a number of advantages to the production field, such as mass production, minimal material waste, the ability to form complex geometries, minimal residual stresses, and the elimination of tool wear [16,17,18]. However, the limitations of this manufacturing technique should not be overlooked. These include dimensional inaccuracy, long printing time, inconsistency of post-processing steps between different printing methods, shrinkage between layers, dimensional change owing to printing parameters, and varying properties (physical and surface) of the final product [16,19,20]. A recent systematic review and meta-analysis by Valenti et al. [21] reported the differences between AM and SM in multiple aspects. It owed the heterogeneity of the mechanical properties of AM restorations to the variability of mechanical tests and the composition of materials. However, the study concluded that AM and SM techniques produced restorations with comparable mechanical properties. The exception was that the flexural strength of SM restorations was better than AM, even after aging.

Internal adaptation and marginal fit of AM or SM restorations are crucial factors for the success of the final dental restoration, just like conventionally made restorations [22,23,24,25,26]. Achieving a minimal marginal gap between the restoration and the preparation is one of the goals during restoration fabrication and cementation to ensure adequate periodontal and pulpal responses and excellent cement performance [20,24]. The marginal mismatch at the restoration/tooth interface can cause the cement to dissolve [24,25], allow microleakage, jeopardize the pupal health [22], initiate secondary caries [23,24], as well as promote periodontal inflammation [24,26]. The literature has reported different ranges of acceptable, marginal gaps depending on the cement type used, restorative material, and measuring technique. However, there is no clear consensus. Marginal opening within 50–120 µm is currently considered acceptable from a clinical perspective for indirectly manufactured restorations; nonetheless, a marginal gap of less than 25 µm would be optimal [20,24,27,28].

One adequate and recommended gap-measuring technique has not been established yet. A variety of options exist, each with its own advantages and limitations. These include [27,29,30,31]: (1) Direct microscopic examination of the marginal area, (2) Measuring distances using cross-sections of the cemented restorations using optical or scanning electron microscopes, (3) Evaluation of light body silicone replicas of the cement gap using a microscope, (4) Laser videography of the digitized silicone replica and die, (5) Indirect measurement of absolute marginal discrepancy using a profilometer, (6) X-ray microtomography, (7) Triple scan method of the restoration, the preparation, and the prosthesis at the try-in stage and super-imposition, and (8) Optical coherence tomography.

On the other hand, trueness refers to the deviations/approximation of the measured values from the intended or planned values, while precision refers to the repeatability of measurement [32]. A greater number of studies investigated the trueness and precision of milled restorations [32,33,34,35,36] compared to those that investigated the same variables for printed restorations [9,15]. Therefore, At the current time, there is no clear decision on whether 3D-printed zirconia crowns can be considered as precise and accurate as the milled ones or not.

Because the AM (3D-printing) of ceramic dental restorations is still in its early stages, there is only a limited number of studies evaluating the restoration adaptation, precision, and trueness of AM full-contour monolithic zirconia crowns. Therefore, this study aimed to investigate and compare the above-mentioned properties of additively manufactured 3Y-TZP crowns compared to the subtractively made counterparts. The null hypothesis stated that internal fit, marginal adaptation/gap, level of trueness, and precision between milled and 3D-printed monolithic zirconia single crowns would be similar.

## 2. Materials and Methods

Based on previous studies [9,15,20,37], a total of 20 monolithic zirconia crowns were made using additive and subtractive manufacturing techniques (10 3D-printed and 10 milled).

A dentate mandibular typodont (Basic study model, KAVO) was used to prepare a mandibular left first molar tooth to receive a monolithic zirconia crown. The preparation configuration and dimensions are listed in Table 1. The preparation was scanned using a 3Shape desktop scanner (3Shape E3 Dental Lab scanner, Copenhagen, Denmark). The acquired Standard Tessellation Language file (STL) was printed into resin (NextDent C&B MFH, NextDent, Soesterberg, The Netherlands) after adding four (2 mm in diameter and height) reference buttons 90 degrees apart around the die at a 2 mm sub-marginal area. The resulting die served as a reference die during fit evaluation.

Additionally, the STL file of this 3D acquisition was used to design a monolithic crown using CAD software (3Shape, Copenhagen, Denmark). The parameters of the crown are shown in Table 1. The CAD file of this crown was saved in STL format and then sent to a 5-axis milling machine (PrograMill PM7, Ivoclar Vivadent, Schaan, Liechtenstein) for subtractive manufacturing using Copran^®^ Zri zirconia disc, shade A1 (Whitepeaks Dental Solutions GmbH, Wesel, Germany) and to a commercial lab for the additive production of crowns using ceramic 3D-printer (CERAMAKER C900 Flex, 3DCeram Sinto, Bonnac-la-Côte, France) and zirconia paste (3DMix ZrO_2_, 3DCeram Sinto, France). In total, 20 crowns were produced (10 per manufacturing process) and sintered following the manufacturer’s directions. Once fired, they were analyzed as received without further treatment.

**Figure 1:**
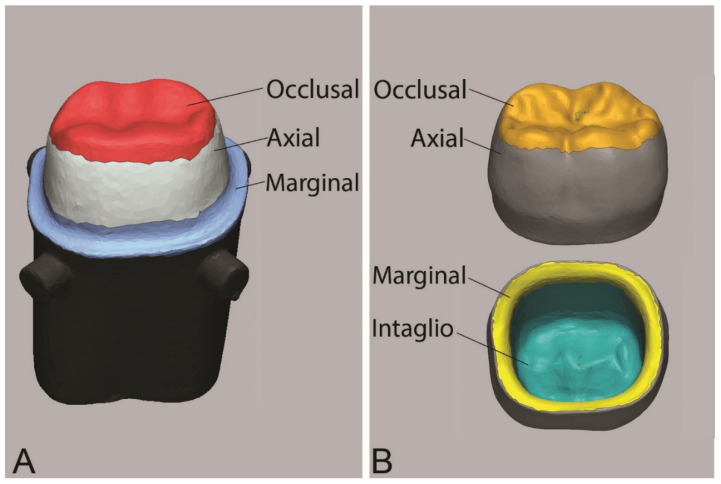
(**A**) Areas of comparison for internal fit and marginal adaptation evaluation, (**B**) Areas of comparison for trueness and precision evaluation.

The master die with the four reference markers was solely scanned initially using 3Shape TRIOS 3 scanner (3Shape, Copenhagen, Denmark), and the resulting file was denoted as a “reference scan.” A micro-brush was used to apply a small amount of polyvinyl siloxane impression adhesive to the occlusal surface of the master die. Following that, the intaglio surface of the crown was lightly lubricated and filled halfway with fit checker material (FIT CHECKER™ ADVANCED BLUE, GC America Inc., Alsip, IL, USA). The crowns were loaded with (49.05 N) until the material set. Then, the excess fit checker was removed with a sharp #15 blade. The crown was retrieved, leaving the fit checker attached to the master die. The master die was scanned again with the fit checker using the same scanner, and the resulting file was denoted “fit scan.” The STL files of the “fit scan” and “reference scan” were imported into Geomagic Control X software, V 2018, (3D Systems Inc., Rock Hill, SC, USA). The “reference scan” was divided into four comparison areas: occlusal, axial (between occluso-axial line angles and medial borders of marginal area), marginal (extending 1.0 mm medially from the finish line), and overall (Figure 1A). The processes of “Initial alignment” and “Best Fit Alignment” between the “reference scan” and “fit scan” were completed. Several cross-sectional views were generated to ensure correct alignment. Then, the process “3D Compare” for the four comparison areas was operated with a color bar range and tolerance of ±0.12 mm. The results report was generated, and the “+ve average” value was considered as the fit value of the crown, which represents the cement gap.

The ISO standard #12836:2015 [38] was followed to evaluate the precision and trueness of the crowns. Each crown was scanned in the following sequence: (1) attachment of putty to the occlusal surface for stabilization, (2) scanning of the intaglio surface of the crown (including margins, inner axial, and inner occlusal surfaces), (3) inversion of putty location into the intaglio surface, and (4) scanning of the cameo surface of the crown. This resulted in a 3D representation of the crown in STL format, denoted as “measured data”.

For the trueness evaluation, the “measured data” scan of each crown was overlayed on the CAD design to measure the deviation from the plan in different areas as follows: occlusal, axial, marginal, intaglio, and overall (Figure 1B). The Root means square (RMS) value was calculated following the methodology described in the literature [39]. RMS represented the deviation of the “measured data” from the original crown design and was presented on a scale of values. The higher the RMS, the lower the trueness is.

For the precision evaluation, the “measured data” scan of the first crown of each experimental group was superimposed on the “measured data” scans of the remaining crowns. The RMS value represents the deviation of the “measured data” from one crown to another. Similarly, the higher the RMS value, the less precise the production method is.

JMP^®^ Software (JMP^®^, Version 16. SAS Institute Inc., Cary, NC, USA, 1989–2022) was used for statistical analysis. T-test was employed to compare differences in means between the two test groups (additive & subtractive) in terms of internal fit and marginal adaptation, precision, and trueness. The level of significance was set to 0.05.

## 3. Results

**Figure 2:**
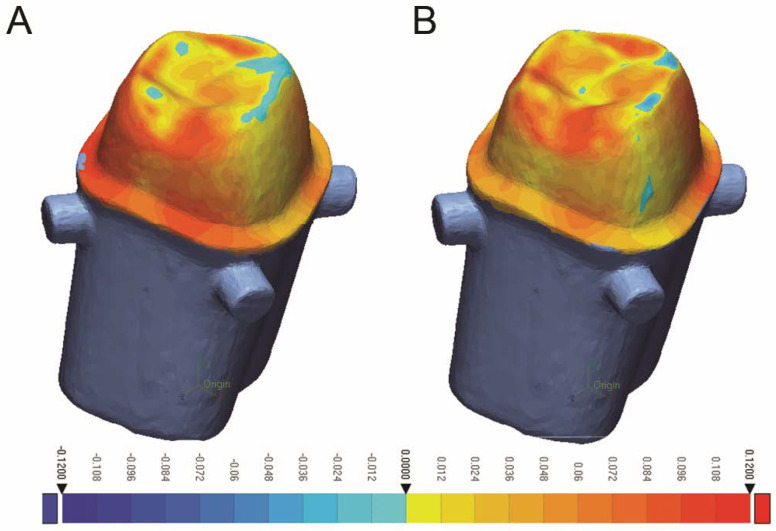
Colorimetric map of fit analysis for (**A**) Milled crowns, (**B**) 3D-printed crowns.

The results revealed no significant difference (*p* > 0.05) between 3D-printed and milled crowns (Table 2) for any of the measured areas for internal fit and marginal adaptation, Figure 2. The axial area showed the highest gap for both groups (milled; 49.23 ± 5.2 µm, 3D-printed; 47.75 ± 3.1 µm).

**Figure 3:**
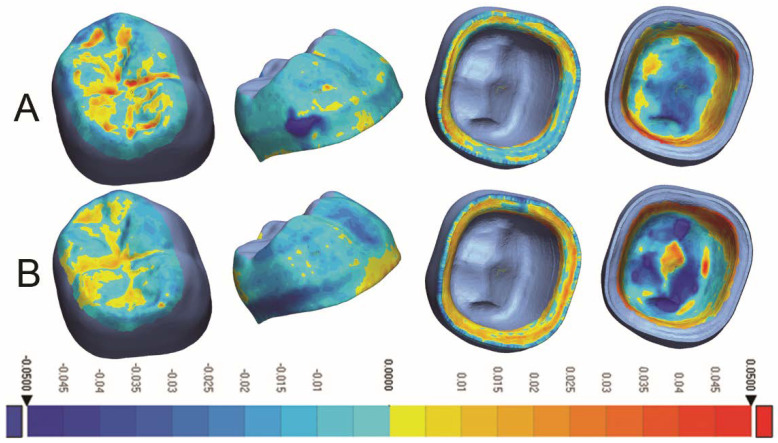
Colorimetric analysis of trueness for (**A**) Milled crowns and (**B**) 3D-Printed crowns.

With regards to trueness evaluation, descriptive statistics are summarized in Table 3. A significant difference was found between the two test groups in three different areas; (occlusal (*p* < 0.0001), axial (*p* = 0.0021), and intaglio (*p* = 0.0130)). The trueness in the occlusal and axial surfaces was better in the 3D-printed crowns, while the trueness of the intaglio surface was better in the milled crowns. No trueness difference was detected in the marginal and overall comparisons (*p* > 0.05), Figure 3.

**Figure 4:**
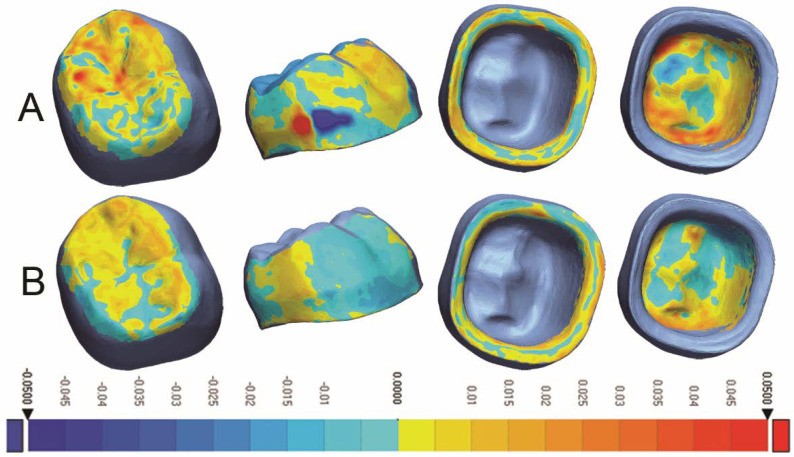
Colorimetric analysis of precision for (**A**) Milled crowns and (**B**) 3D-Printed crowns.

Descriptive statistics for precision evaluation are summarized in Table 4. Statistical analysis showed a significant difference in precision between test groups in all areas (occlusal, axial, marginal, intaglio, and overall (*p* < 0.001)), with 3D-printed crowns revealing better precision compared to the milled ones, Figure 4.

## 4. Discussion

This study investigated the 3-dimensional internal fit and marginal adaptation of monolithic 3D-printed and milled zirconia crowns. Additionally, the study evaluated the level of trueness and precision of the produced crowns compared to the original design and to each other. The results of the study suggest partial acceptance of the null hypothesis with regard to adaptation, while it was rejected in relation to trueness and precision.

In this study, the specimens were made using two manufacturing techniques (additive and subtractive) using the same STL file to ensure consistency and standardization of the produced crowns. Additionally, the digital method (3D-superimposition) to analyze the fit and adaptation allows complete visualization of the intaglio surface and measurement of a huge number of points (making up the whole surface area of the intaglio side) compared to selected point measurements.

Crown fit and marginal adaptation are affected by a number of factors, including the manufacturing system and technique, processing parameters, the raw material, preparation design, shrinkage upon sintering, and the cement used, among others [20,40]. In the current study, the fit and adaptation of both types of crowns were comparable to each other regardless of the measured area. The findings for milled and 3D-printed crowns (marginal ~37–38 µm and overall ~45–47 µm) were still within the acceptable range reported in the literature (50–120 µm) [20,24,27,28]. The marginal gaps (extending between the finish line to 1 mm medially) showed the smallest values in both groups, confirming the effect of the minimal cement spacer incorporated in that area. Additionally, the difference in values between the two groups might indicate a similar sintering shrinkage. Thus, these findings were not in alignment with multiple studies [41,42,43] that reported values for marginal, axial, and occlusal distances between 42–159 µm. Thus, in these studies, the silicon replica method was utilized to measure solitary points around the margin or at the occlusal surface.

The findings of this study concurred with other reports in the literature regardless of the variation in materials used, measuring time, location, and technique. In a systematic review [27] of CAD/CAM-made all-ceramic restorations (not exclusive for zirconia) and based on 54 in vivo and in vitro studies, the average marginal gap was 120 µm with a range of 3.7–174 µm. The significant heterogeneity in the reported values was linked to a large number of systems and materials used and the discrepancies among studies in measuring techniques. Freire et al. [44] reported a marginal gap of 31.0–47.4 µm depending on the initial scanning method (intraoral or extraoral scanning). Another study [45] based on measuring the marginal discrepancy at eight circumferential points around monolithic zirconia crowns fabricated from pre-sintered blocks reported a range of 38–60 µm based on measuring stage (post-sintering, post-glazing, and post-cementation). Additionally, production parameters (finish line, crown thickness, and sintering protocols) were also investigated to determine the effect on marginal gaps [46], and the reported values ranged between 11–52 µm for all groups. Thus, all these reports were related to milled zirconia crowns.

As compared to milled zirconia, fewer studies exist on the use of 3D-printed zirconia in dentistry [17,47,48,49,50,51,52], in particular, those that analyzed the marginal gaps of additively fabricated monolithic zirconia crowns [20,53]. Li et al. [53] reported higher values (occlusal 63 µm, axial 135 µm, and marginal 169 µm) compared to our results. Similarly, Revilla-León et al. [20] found larger median gaps in marginal (146 µm) and internal (79 µm) areas than seen in this study. The variation between these studies and ours might be linked to the materials used or the measuring method. In the former study [53], a different 3D printer (CSL 150, Porimy, Kunshan, China) and intraoral scanner (CEREC Omnicam, Dentsply Sirona, Charlotte, NC, USA) were used with no mention of the zirconia paste brand. While in the latter study [20], the zirconia paste (3DMix ZrO_2_, 3DCeram) and printer (CERAMAKER C900, 3DCeram) matched those used in our study but the measuring technique involved the use of silicon replica.

High trueness and precision of the final crown as compared to the designed restoration is not only important for adequate crown fit on the prepared tooth but also to minimize chair-side corrections and modifications of the restoration [54]. The trueness of an object refers to the correspondence of the measured points of the specimen with that of the plan/design. In our study, 3D-printed specimens showed better trueness and closer representation of the original design on the cameo surface of the crown, while the milled crowns had better trueness in the intaglio surface. Marginal trueness was comparable in both manufacturing techniques. Regardless of the trueness discrepancies between the two manufacturing techniques in multiple measured areas, the overall trueness of both types of crowns was similar. The greater deviation of the occlusal surface of milled crowns might be related to the number of milling burs and axes or the geometric and diameter limitations of the milling burs [33]. The trueness findings of this study do not match those reported in the literature [9,15,37,55,56]. The discrepancies detected could be related to differences in the analysis method utilized (absolute average, RMS, or (90°/10°)/2) [15], scanning device [15,37,55], the use of powder spray prior to scanning [9,37], or the number and orientation of support structure during production [56].

Dental restorations must be produced with high precision to ensure proper fit and adequate biological response. The precision of a printed object is dependent on multiple factors, including the printing technique, the number, location, and size of supports, and the digitizing method/scanner [57]. The authors of this study aimed to check the precision of the produced crowns per manufacturing technique. Therefore, the “measured data” of one crown was compared to that of the rest of the crowns within each manufacturing technique. The precision of the additively made crowns was better in all evaluated areas compared to milled crowns. This might be related to aspects of the manufacturing process. All 3D-printed crowns were made simultaneously using the same zirconia paste/printer and production steps. On the other hand, milled crowns were manufactured consecutively using the same zirconia disc. The subtractive manufacturing procedure induces changes in the milling burs, starting after the first mill [58]. This eventually affects the trueness of the produced restoration and the precision thereafter. Additionally, the position of the zirconia crown within the disc and its relation to the milling bur and spindle is not consistent among the 10 produced crowns, which may increase the deviation from the CAD file [32]. The precision has been reported by other studies to be comparable in both manufacturing techniques [15]. However, the measurement in that study involved evaluation by calibrated prosthodontist to evaluate the proximal contact and marginal area.

Among the limitations of the study is the use of a single brand/material in each manufacturing technique. Moreover, the inconsistency between the published literature with regard to the distribution and extent of measured areas (occlusal, axial, marginal, internal, external, etc.) made the comparison with previous studies more challenging. Additionally, specimens were tested as sintered and were not subjected to cyclic fatigue that could show different types of marginal adaptation [59]. Therefore, the authors recommend testing more brands of 3D-printed zirconia crowns and different additive manufacturing techniques. In addition, it is recommended to create a standardized distribution of measuring areas and to correlate the effect of bur deterioration to crown trueness and precision, in addition to evaluation of marginal discrepancy of aged crowns.

## 5. Conclusions

Within the limitations of this in vitro study, it can be concluded that:

1. Additive and subtractive manufacturing techniques produced zirconia crowns with comparable internal fit and marginal adaptation.
2. The 3D-printed crowns had better occlusal and axial trueness, while milled crowns showed better intaglio trueness.
3. 3D printing produced more precise restorations than milling.
4. Further in vitro and in vivo studies are needed to evaluate other aspects of 3D-printed zirconia crowns, such as mechanical and optical properties.

## Figures and Tables

**Table 1:** Preparation features and crown parameters.

Preparation Feature	Amount	Crown Parameters	Amount
Anatomic occlusal reduction	1.5–2.0 mm	Cement gap	60 µm
Axial reduction	0.8–1.2 mm	Extra cement gap	40 µm, 1 mm from margin
Finish line	0.8 mm shoulder with rounded internal line angle	Offset angle	55°
Total occlusal convergence	8–10°	Offset extension	0.10 mm

**Table 2:** Marginal gap and internal fit mean ± standard deviations (SD) at four different measurement areas (µm).

Measured Area	Milled Mean Gap ± SD	3D-Printed Mean Gap ± SD	*p*-Value
Occlusal	40.20 ± 7.96	45.67 ± 4.57	0.0756
Axial	49.23 ± 5.25	47.75 ± 3.16	0.4545
Marginal	36.68 ± 6.04	38.26 ± 4.87	0.5277
Overall	44.63 ± 6.24	46.67 ± 2.80	0.3581

**Table 3:** Trueness means RMS ± SD (µm) and significance values between the milled and 3D-printed crowns.

Measured Area	Milled RMS ± SD	3D-Printed RMS ± SD	*p*-Value
Occlusal	14.78 ± 2.23	8.77 ± 0.89	<0.0001 *
Axial	20.37 ± 4.49	14.77 ± 2.03	0.0021 *
Marginal	16.24 ± 4.62	16.35 ± 0.84	0.9417
Intaglio	20.29 ± 3.82	23.90 ± 1.60	0.0130 *
Overall	18.58 ± 3.03	17.00 ± 0.95	0.1329

* Indicates a significant difference between the test groups per measured area.

**Table 4:** Precision means RMS ± SD (µm), and significance values between the milled and 3D-printed crowns.

Measured Area	(Milled) RMS ± SD	(3D-Printed) RMS ± SD	*p*-Value
Occlusal	13.30 ± 2.46	7.82 ± 1.06	<0.0001 *
Axial	20.76 ± 5.62	9.60 ± 1.84	<0.0001 *
Marginal	16.84 ± 3.94	9.73 ± 0.92	<0.0001 *
Intaglio	15.72 ± 2.96	10.68 ± 1.22	0.0002 *
Overall	17.31 ± 3.39	9.59 ± 0.75	<0.0001 *

* Indicates a significant difference between the test groups per measured area.

## Data Availability

The data can be provided by corresponding author upon reasonable request.
